# An integrative network approach for longitudinal stratification in Parkinson’s disease

**DOI:** 10.1371/journal.pcbi.1012857

**Published:** 2025-03-28

**Authors:** Barry Ryan, Riccardo Marioni, T. Ian Simpson

**Affiliations:** 1 School of Informatics, The University of Edinburgh, Edinburgh, United Kingdom; 2 Centre for Genomic and Experimental Medicine, Institute of Genetics and Cancer, The University of Edinburgh, Edinburgh, United Kingdom; University of Wisconsin, Madison, UNITED STATES OF AMERICA

## Abstract

Parkinson’s disease (PD) is a neurodegenerative disorder characterized by motor symptoms resulting from the loss of dopamine-producing neurons in the brain. Currently, there is no cure for the disease which is in part due to the heterogeneity in patient symptoms, trajectories and manifestations. There is a known genetic component of PD and genomic datasets have helped to uncover some aspects of the disease. Understanding the longitudinal variability of PD is essential as it has been theorised that there are different triggers and underlying disease mechanisms at different points during disease progression. In this paper, we perform longitudinal and cross-sectional experiments to identify which data modalities or combinations of modalities are informative at different time points. We use clinical, genomic, and proteomic data from the Parkinson’s Progression Markers Initiative. We validate the importance of flexible data integration by highlighting the varying combinations of data modalities for optimal stratification at different disease stages in idiopathic PD. We show there is a shared signal in the DNAm signatures of participants with a mutation in a causal gene of PD and participants with idiopathic PD. We also show that integration of SNPs and DNAm data modalities has potential for use as an early diagnostic tool for individuals with a genetic cause of PD.

## Introduction

Parkinson’s disease (PD) is a heterogenous, progressive, multisystem neurological disorder that affects the nervous system. It is most commonly characterised by a range of motor symptoms, primarily involving diﬃculties with movement, however a wide variety of non-motor symptoms also exist. PD has a complex pathophysiology, but these disease pathways culminate in the gradual death of neuronal cells, causing a deficit in dopamine [1].

One notable aspect of PD is the variability between individuals with the disease. PD is characterised by core motor syndromes of tremor, rigidity, bradykinesia and postural instability. The onset, trajectory and experience of these symptoms among people varies greatly. For example, some patients experience a rapid progression to disability and others following a relatively benign course [2]. While PD medications do not cure the disease, they do help with some of the day-to-day motor symptoms, however the time period for which they are effective varies between patients also [3].

Identification of mutations in single genes have aided the understanding of PD with approximately 30% of cases being attributed to a patients’ genetics [4]. For example, specific variants in the *LRRK2*, *GBA*, and *PINK1* genes are associated with PD [3]. This motivates the use of omic measures for uncovering novel insights into the pathology of PD. Omic data modalities capture genetic and/or biomolecular profiles; analyses of these data has resulted in many novel findings in PD. Craig et al. (2021) found early alterations between the gene expression of PD patients and healthy individuals [5]. Similarly, Kern et al. (2021) found that non coding RNA’s can have diagnostic and prognostic power in PD individuals [6]. These mutations in single genes are not 100% penetrative, and despite these advances, recent Genome Wide Association Studies of PD have had conflicting results. Walters et al. (2023) found no genome wide significant loci for PD in the China Kandoori Biobank with a population of 105,408 Chinese individuals [7]. Conversely, in a population of 2478 Chinese individuals, Pan et al. (2023) found 19 associations with PD including genome wide significant loci in *LRRK2*, *SNCA*, and *GBA* [8]. Currently, there is no known exogenous or genetic trigger for all PD patients that causally results in the loss of dopaminergic cells.

An explanation that has been hypothesised is that the disease mechanisms of PD change over time [1]. Longitudinal variability poses significant challenges in both the biological understanding and treatment of PD. This heterogeneity necessitates a flexible approach that can incorporate multiple sources of information at a given stage of PD. The Parkinson’s Progression Markers Initiative was created for this reason. It consists of longitudinal clinical, genomic, and imaging data from over 900 PD cases, 800 Prodromal (PL) (cases without a clinical diagnosis for PD, but early indicators that they will go on to develop it) and 230 Healthy Control (HC).

We propose a flexible integrative approach which can include both genomic modalities and the longitudinal component of the PPMI dataset. Previous multi-modal research has achieved good prediction accuracy using the PPMI dataset. Chan et al. (2022) achieve perfect disease stratification using a model which incorporated multiple omic and image datasets [9]. A review by Gerraty et al. (2023), on multi-modal integration approaches in the PPMI dataset, found that clinical and neuroimaging datasets were the most commonly used modalities [10]. They further identified that few machine learning focused papers use the longitudinal structure of the PPMI study. A possible reason for this is due to restricted patient coverage when incorporating image data. Chan et al. identify that the dataset they utilised is small and heavily skewed to PD patients [9]. Given the time-consuming nature and expense of collecting image data, this is not surprising.

In this analysis, we integrate omic datasets such as mRNA and SNPs with clinical and proteomic information using a flexible network taxonomy that allows retention of the maximum number of patients. We represent the integrated modalities as a Patient Similarity Network (PSN) and use an Graph Convolutional Network (GCN) architecture for disease stratification. We group patients into those with a mutation in a known causal gene for PD, those who have a sporadic onset of the disease, and finally a combination of both. In each case we attempt to classify individuals as either having PD, being PL, or a HC. We perform experiments cross-sectionally across 4 time points over the course of the first three years of a patient’s disease post diagnosis. We assess the best combination of modalities at each time point and contrast the findings between the three groups. Finally, we train longitudinal models on a subset of genetic PD patients who have data at all time points. The goal of this experiment is to assess if the learnt disease signatures remain consistent across the 4 time points.

## 1 Methods

### Multi-Omic Graph Diagnosis (MOGDx)

MOGDx, shown in S1 Fig, is a flexible tool to integrate multiple omic measures and perform classification tasks. It uses a patient similarity measure to identify patients who have similar molecular, epigenetic, and demographic disease characteristics and performs node classification using a GCN. The performance of MOGDx was benchmarked on cancer data and achieved state-of-the-art performance compared to similar research [11].

There are two main components in the MOGDx framework; PSN generation and Graph Convolutional Network with Multi-Modal Encoder training and classification. A single PSN is built per modality. Feature selection is performed, and similarity is measured between these features using Pearson correlation, where suitable, otherwise Euclidean distance. Each PSN is constructed from the similarity matrix using the k nearest neighbours algorithm. Similarity Network Fusion (SNF) is then used to combine individual PSN’s into a single network. As is common practice, all patient information is used to construct the network, with train, validation and test labels created during the training phase of the GCN-MME [12, 13]. In this approach, non-informative features are discarded during the similarity calculation to discourage uniform similarity scores for modalities such as DNAm which will have a large number of redundant or similar features.

The fused PSN and the modalities are input into the GCN-MME for training and classification. Each modality is compressed using a two layer encoder. The first layer of the encoder is of fixed length, with the second layer being tuned to each modality by performing a hyperparameter search. The compressed encoded layer of each modality is then decompressed to a shared latent space using mean pooling. This methodology follows similar encoder architectures established in other works [14, 15]. Median imputation is also performed at the second layer of each encoder to retain patients if they are missing from that modality. The shared latent space corresponds to the patient node features, which are combined with the PSN and input into the GCN for classification. The GCN-MME is trained under the semi-supervised setting for graph neural networks outlined by Hamilton (2020) [16]. For a detailed description of the MOGDx architecture, please refer to Ryan et al. 2023 [11].

MOGDx is a suitable tool to perform analysis on the PPMI dataset due to its flexibility. It can integrate any number of modalities, whilst simultaneously retaining the maximum number of patients possible, in contrast to other existing methodologies. As discussed by Chan et al. (2022) and as per Table 1, there are fewer HC participants [9]. Moreover, not every participant will be present in each modality at each time point. In order to avail of the full PPMI dataset, a method which can incorporate the maximum number of participants is required. MOGDx achieves this by utilising SNF and imputation to retain patient nodes without a large degradation in performance [11]. MOGDx also provides a high level of interpretability. Due to the flexibility of integration, ablation experiments can be performed to identify the most predictive modalities. As feature selection is performed in the MOGDx pipeline, these features can be further analysed to identify important pathways, traits or interactions of the target application.

### PPMI dataset

Data was obtained from the publicly available PPMI dataset [17]. The participant count per year, modalities analysed and number of raw features per modality are summarised in Table 1. In total, 6307 samples from 2188 participants were included in the analysis. Participants in the analysis were identified as PD, PL or HC and by disease subtype of genetic, idiopathic, Rapid eye movement Behaviour Disorder or hyposmia.

**Table 1:** Breakdown of PPMI dataset by number of participant and modality features.

	Parkinson’s Disease (PD)		Prodromal (PL)			Healthy Control (HC)	Total
	Genetic	Idiopathic	Genetic	RBD	Hyposmia		
Participant Count							
Year 0	465	631	633	97	91	270	2187
Year 1	244	448	423	50	35	203	1403
Year 2	282	365	705	36	22	180	1590
Year 3	198	360	348	36	19	166	1127
Total	1189	1804	2109	219	167	819	6307
**Modality**	**Raw Feature Count**		**Feature Count post Processing**			**Method of Feature Selection**	
			**Genetic + Idiopathic**	**Genetic**	**Idiopathic**		
mRNA	52338		29791	24251	33664	$p_{adj}<0.05$	
miRNA	40194		3206	2995	3152	$p_{adj}<0.05$	
SNP	841		20	20	20	None	
DNAm	805434		300k	300k	300k	| *ω* | > 0	
Protein	4785		4785	227	4785	| *ω* | > 0	
Clinical	6		6	6	6	None	
MDS-UPDRS	88		63	63	63	| *ω* | > 0	

$p_{adj}$ is the false discovery rate in differential expression.  | *ω* |  is the absolute coeﬃcient weights in penalised elastic net regression.

The four genomic measures analysed, namely Messenger RNA, Micro RNA, DNA Methylation (DNAm) and Single Nucleotide Polymorphisms, were generated from whole-blood samples by PPMI. Each genomic dataset was further processed in a complimentary bioinformatics pipeline, if available. For example, DNAm was normalised using the wateRmelon package in R [18]. See Ryan et al. (2023) for more detail on how specific genomic modalities were handled [11]. In general, processing included the removal of zero variance or missing features, normalisation, imputation of missing values and conversion from categorical to numerical features. The top 300k most variable CpG sites were retained to allow computation on this dataset to fit into memory. For the same reason, a principal component analysis was performed on the SNPs dataset to reduce the dimensionality of the dataset, and the first 20 PC’s were retained.

Genomic datasets were supplemented with additional measures of 1472 Cerebral Spinal Fluid (CSF) protein markers extracted from participants and clinical descriptors. Clinical descriptors included individual phenotypes of age, sex and years of education; These were supplemented with measures for smoking, alcohol and BMI generated from DNAm profiles [19]. These DNAm profiles were derived from models trained on up to 5087 individuals in a national study in Scotland and tested on two separate cohorts also based in Scotland [19]. The Movement Disorder Society Unified Parkinson’s Disease Rating Scale by Goetz et al. (2008) is a measure of disease severity in those with PD and PL [20]. This scale combines measures relating to both motor and non-motor symptoms of PD. It consists of both self-assessment and clinical assessments and is a proxy of disease stratification [20]. It was used as a baseline comparative model to identify if the biological signal for PD found in the blood is stronger than clinical assessment using MOGDx. These modalities were similarly processed for feature selection, conversion and normalisation.

As per Table 1, there were two methods for feature selection. Where suitable pairwise linear regression between the three strata was performed using the DESeq2 package in R [21]. In other modalities, penalised elastic net regression was performed using the glmnet package in R [22]. Differentially expressed genes with a statistically significant FDR ($p_{adj}<0.05$) and logistic regression coeﬃcients with an absolute weight greater than zero were used as selected features in the similarity calculation. The number of features selected depended on the subgroup being analysed. If no informative features were found, all features were retained.

Table 1 shows the participant availability per year. The time points cover the first three years of the disease in the PD cohort. The first time point (labelled year 0) corresponds to participants with PD who have had a diagnosis for less than 2 years, have not begun taking any PD medication and are not expected to require PD medication for at least 6 months [17]. Those in the genetic subgroup of PD have a mutation in one of three genes: *LRRK2*, *SNCA* or *GBA*. Idiopathic individuals do not have mutations in any of these three genes. PL participants have been identified as being of high risk for the disease, but have not yet met a clinical threshold for diagnosis. The first time point, year 0, in this cohort corresponds to their enrolment in the study. The genetic PL subgroup also have a mutation in one of the three aforementioned genes as aligned with the genetic PD subgroup. As per Table 1 the PL participants in the genetic subgroup far outnumber the participants in the RBD and hyposmia subgroups. Participants in these groups have one of two non-motor symptoms associated with PD. RBD is a sleep disorder and hyposmia is a smell disorder, both of which have been identified as early indicators for PD [23, 24]. HC participants were screened by PPMI to ensure they did not meet the criteria for either PD or PL. As with PL, their first time point, year 0, aligns with their enrolment in the PPMI study. PD idiopathic and PL genetic are the two most prevalent subgroups in the dataset. As identified in Chan et al. (2022) there are fewer HCs compared to PD and PL however the numbers presented in Table 1 show higher counts compared to their analysis which was subset to participants who had image data available [9]. A distinguishing factor of this analysis is the utilisation of the longitudinal data in the PPMI dataset. As per Table 1, over time, the number of participants decreases across all strata and subgroups. This is in part due to participant dropout (n = 401), missing samples for a participant at a time point or the transition from PL to a clinical diagnosis for PD (n = 33). Summary of the criteria for participant stratification and disease subgroups are summarised in S2 Fig.

### Cross-sectional and longitudinal experiment design

**Fig 1:**
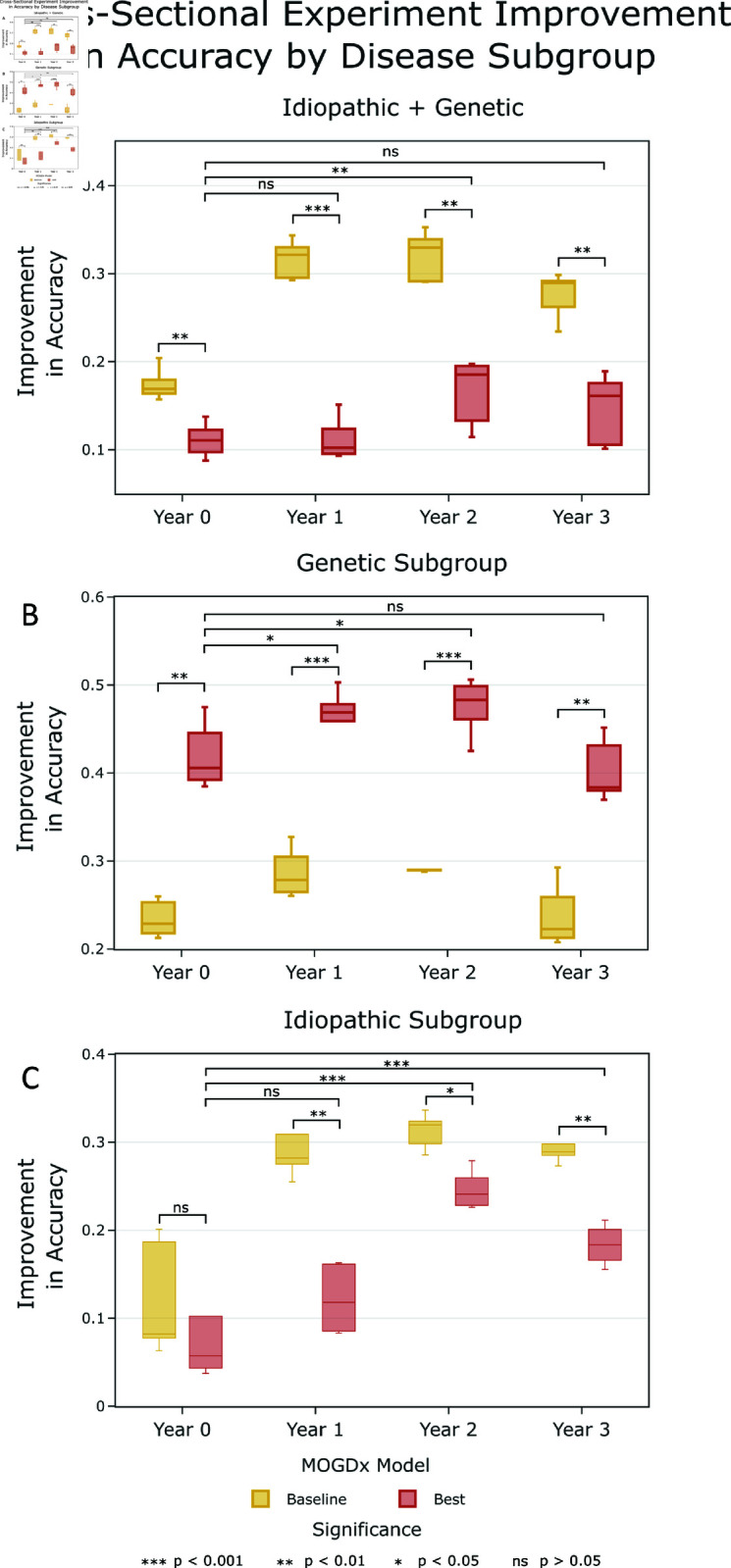
Performance of Best vs. Baseline (MDS-UPDRS) models at each time point. (A) Idiopathic & Genetic, (B) Genetic, (C) Idiopathic. The best model in each subgroup at each time point corresponds to the combination of modalities reported in Table 2. The performance of the MDS-UPDRS baseline models are reported in S1 File.

In this analysis, we perform both cross-sectional and longitudinal experiments at 4 time points over three years. In all experiments we classify whether participants have PD, are PL or are a HC. We perform cross-sectional experiments on all participants, regardless of their subgroup and on two subsets based on participants’ subgroup. The first subset, referred to as genetic, includes PD and PL participants in the genetic subgroup. The other subset, referred to as idiopathic, includes participants in the idiopathic PD, RBD and hyposmia subgroups. HC participants are included in both subsets as a control. We use a brute-force approach, testing all combinations of modalities in each experiment to identify the modalities at each time point with the highest accuracies and F1 scores. For fairness of comparison, we train each model independently and perform cross-validation on each model to obtain standard errors for each models’ performance. We also account for differences in the number of participants by using F1-score and comparing each model to a baseline, which only predicts the most common class. In this manner, we can fairly attribute improved performance due to the inclusion or exclusion of a modality. Where two models achieve comparable performance, we report the model which includes the greatest number of participants. The top performing model at each time point, per subgroup is reported in Table 2 and shown in Fig 1 with all model’s performance reported in S1 File.

**Table 2:** Cross-sectional performance of MOGDx in different subgroup experiments.

		Modalities	Number of Participants	Accuracy	F1 score	Improvement in Accuracy
Genetic + Idiopathic (All)	Year 0	DNAm + SNP + mRNA + miRNA	1515	0 . 630 ± 0 . 019	0 . 665 ± 0 . 017	0 . 110 ± 0 . 018
	Year 1	DNAm	548	0 . 624 ± 0 . 020	0 . 667 ± 0 . 032	0 . 111 ± 0 . 02
	Year 2	Clinical + DNAm	542	0 . 694 ± 0 . 037	0 . 717 ± 0 . 034	0 . 166 ± 0 . 037
	Year 3	DNAm	493	0 . 712 ± 0 . 018	0 . 699 ± 0 . 048	0 . 146 ± 0 . 018
Genetic	Year 0	DNAm + SNP	489	0 . 789 ± 0 . 036	0 . 753 ± 0 . 04	0 . 419 ± 0 . 036
	Year 1	DNAm + SNP	443	0 . 867 ± 0 . 018	0 . 835 ± 0 . 02	0 . 472 ± 0 . 018
	Year 2	DNAm + SNP	432	0 . 866 ± 0 . 031	0 . 837 ± 0 . 032	0 . 477 ± 0 . 031
	Year 3	DNAm + SNP	365	0 . 841 ± 0 . 034	0 . 811 ± 0 . 038	0 . 403 ± 0 . 034
Idiopathic	Year 0	SNP + miRNA	667	0 . 681 ± 0 . 031	0 . 752 ± 0 . 008	0 . 069 ± 0 . 031
	Year 1	CSF + DNAm + SNP	582	0 . 720 ± 0 . 039	0 . 776 ± 0 . 035	0 . 122 ± 0 . 039
	Year 2	CSF + Clinical + DNAm	399	0 . 805 ± 0 . 022	0 . 770 ± 0 . 022	0 . 246 ± 0 . 022
	Year 3	CSF + DNAm	360	0 . 764 ± 0 . 022	0 . 721 ± 0 . 021	0 . 183 ± 0 . 022

In the longitudinal analysis, we repeat the best performing cross-sectional experiment on the genetic PD (n = 70) and genetic PL (n = 84) subgroups. Once again, HC (n = 150) are included as a control. This analysis is restricted to participants who are present at each time point in at least one of the included modalities. Only the optimal combination of modalities which maximised both accuracy and patient retention was analysed. This was identified by averaging the F1 scores of each cross-sectional model across all time points. A model integrating these modalities is trained and tested at each time point. These models are then tested on unseen networks and modalities of the same patients but from the alternative time points. Participants were split into training and test sets, thus performance for all models at all time points are reported on the same test set. Networks were reconstructed during this phase using only the features selected at the test time point. For example, when testing the year 3 network at year 1, the network being tested is reconstructed only using the features selected at year 1. This is undertaken to assess if the biological signal learnt at one time point is present at other time points.

## 2 Results

### Performance and evaluation

The performance metrics used to compare the classification performance of MOGDx were accuracy, F1 score and improvement in accuracy. The F1 score was calculated by the mean F1 score of each class, weighted by the size of that class. Improvement in accuracy is a metric used to compare how much the accuracy improved compared to a baseline model which only predicts the most common class. Stratified k-fold cross validation was performed with 5 randomly generated splits to obtain the mean and standard deviation metrics reported. Within each split, the training set was further randomly split into training and validation sets to produce an overall train/validation/test split of 68%/12%/20% respectively.

### A multi-modal approach is optimal when stratifying individuals with PD over time

The optimal combination of modalities at each time point in each subgroup are reported in Table 2 and shown in Fig 1 as the best model. Confusion matrices are also provided in S3–S5 Figs for multi-modal performance assessment. The results from the cross-sectional experiments favour a multi-modal approach. The power of flexible integration when stratifying participants in the PPMI dataset with PD is apparent as all 6 modalities are included in at least one experiment. In Table 2, only two experiments, years 1 and 3 with all participants (genetic + idiopathic), do not integrate modalities. In Fig 1, performance in the combined genetic + idiopathic and in the idiopathic subgroup alone are significantly poorer than the MDS-UPDRS baseline model. Only the genetic subgroup achieves a consistent improvement in accuracy greater than the MDS-UPDRS assessment. This could motivate the use of these modalities for early disease diagnosis, as motivated below. The combination of CSF and DNAm in the idiopathic subgroup shows promising performance, particularly later in the disease course. Comparing to a model trained on the MDS-UPDRS assessment is a diﬃcult task, given it consists of clinical assessment scores of both motor and non-motor symptoms [20]. Thus, the results show encouraging performance when integrating combinations of modalities in subgroups of PD.

### Disease signatures for PD can be learnt from whole-blood samples and protein markers in PPMI study participants

Table 2 shows there is an improvement in accuracy compared to a model which predicts the most common class in all experiments. This improvement increases with time, indicating an increased biological signal for PD as the disease progresses. In both genetic and idiopathic subgroups, years 1 and 2 are the most predictive time points. This could indicate that these time points are capturing both early and late signatures of PD. This is particularly evident in the idiopathic subgroup, where there is a change in predictive modalities over time, with common modalities early and late in the disease. The SNPs modality is predictive early, whereas protein CSF markers along with DNAm are more prominent in later stages. This supports the work of Wüllner et al. (2023) who found that there may be different disease mechanisms at different stages of PD and highlights the necessity of flexible modality integration [1]. The caveat is that the prediction accuracy at year 0 in this subgroup is low.

Conversely, in the genetic subgroup, the modalities which are most predictive do not change with time. SNPs are a fixed description of participant’s genetic status [25] and the sub-population of participants that carry pathogenic mutations with varying penetrance in the PD and PL groups are likely to share other features across their genomes that influence the extent and nature of the onset of PD symptoms. The ability of SNPs derived signatures alone to distinguish between PD/PL and HC participants in our study but not between PD and PL participants supports this idea. We demonstrate that this further separation can be achieved by inclusion of an additional modality, in this case DNA methylation. Whether the biological signal learnt changes over time requires further work to understand the drivers of variability in DNAm at each time point. The integration of these two modalities outperforms the MDS-UPDRS baseline, highlighting the predictive power of using whole-blood samples to extract genomic information relating to a neurological disease. Overall, we show there are disease signatures early, but particularly late, in the blood of individuals with PD, despite it being a neurological disorder.

### There are possible shared DNAm signatures in the idiopathic and genetic disease subgroups of PD

As per Table 2, there is a clear genetic driver in participants who have a mutation in a causal gene of PD. This highlights the homogeneity between participants in this subgroup. Conversely, the idiopathic cohort is heterogenous, as this group combines participants with unknown and likely different causes of PD. Despite these significant differences between participant subgroups, most experiments include DNAm as a predictive modality, with the idiopathic subgroup at year 0 being the only experiment where it is not included. Given this prominence, it indicates the presence of epigenetic modifications between PD, PL and HC participants. When considering all participants (genetic + idiopathic), there is a mix of a homogeneous and a heterogeneous group, which makes learning very diﬃcult. Despite this, there is a robust improvement in accuracy between 10% and 20% across all time points, as per Fig 1. This suggests that there may be a shared signal between the two subgroups in the DNAm modality. A possible explanation for the decreased performance compared to the two subgroups is that the additional information added by other integrated modalities is not shared between the two subgroups. In order to confirm if the signal being learnt is similar, more research needs to be conducted to identify the common discriminating DNAm features, however our results suggests common signatures in the DNAm of genetic and idiopathic PD participants.

### An integrative model trained at a late disease stage could form a viable early diagnostic tool for individuals with a genetic predisposition for the disease

In the cross-sectional experiments, we show the metrics for classifying participants with a mutation in a causal gene of PD to be very promising. As per Fig 1 and Table 2, the combination of DNAm and SNPs achieves a consistently high accuracy, F1 score and improvement in accuracy. These results motivate the integration of these modalities for early disease detection. We performed longitudinal experiments on a subset of participants from the genetic group who are present in either DNAm or SNPs at each time point. These experiments were designed to identify the optimal time point to train such a diagnostic tool and if the disease signal learnt early in the disease is present later and vice versa.

Table 3 highlights that an early PD detection model should be trained later in the disease course. Both the accuracy and F1 scores increase with models that are trained at later time points. Optimal performance was observed by the model trained at year 3. Poorest performance was observed by the model trained at year 0, with the performance of models trained at years 1 and 2 being comparable. S6–S9 Figs show the multi-modal performance of each model at each time point. These figures highlight the improved discrimination between PD and PL participants for models trained later in the disease course.

**Table 3:** Longitudinal experiments performance metrics.

Accuracy / F1		Time Point Model Tested			
		Year 0	Year 1	Year 2	Year 3
Time	Year 0	0 . 816 / 0 . 785	0 . 803 / 0 . 774	0 . 816 / 0 . 781	0 . 803 / 0 . 769
Point	Year 1	0 . 855 / 0 . 839	0 . 908 / 0 . 882	0 . 829 / 0 . 794	0 . 816 / 0 . 780
Model	Year 2	0 . 868 / 0 . 844	0 . 789 / 0 . 756	0 . 855 / 0 . 820	0 . 829 / 0 . 800
Trained	Year 3	0 . 921 / 0 . 899	0 . 921 / 0 . 908	0 . 934 / 0 . 916	0 . 974 / 0 . 965

**Fig 2:**
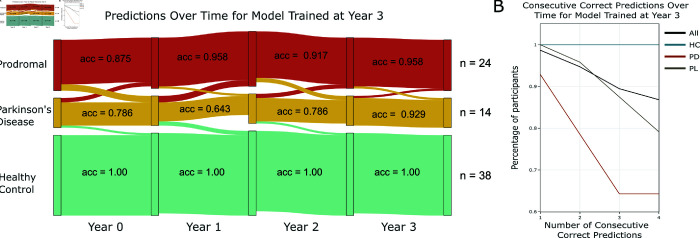
Patient stratification for model trained at year three. (A) Within class accuracy and predictions, with the interconnecting bands showing incorrect model predictions and the thickness of the bands reflecting the number of patients misclassified. (B) The percentage of times the model correctly classified an individual patient. The sharp decline in the PD class shows there is a subset of ˜65% of PD participants who are more easily identified at all time points.

In Fig 2 the within class accuracy is reported at each time point with interconnecting bands showing incorrect model predictions. We can see the model misclassifies more PD patients as PL at earlier time points, as denoted by the thickness of the red band (Fig 2A). We also assessed the longitudinal behaviour of the model to see whether it consistently identified the disease class of participants as PD, PL and HC throughout the time course (Fig 2B). There is a subset of ˜35% PD participants who are the most diﬃcult for the model to classify. This subset only has either 1 or 2 correct classifications, whereas both the PL and HC classes maintain ˜90% accuracy at all time points. The HC class is the easiest to predict. As mentioned, both PD and PL participants have a genetic risk variant for the disease, thus, the SNPs modality can easily discriminate between them and the HC participants. The main differentiation between the models is their ability to distinguish PD from PL participants. As mentioned, the accuracy in predicting PD participants decays the further away in time you test the model from when it was trained. This can be observed both by the sharp gradients of the PD participants when assessing the number of consecutive correct predictions of the model and the decrease in accuracy over time in Fig 2A. Conversely, the PL class have much more stable and consistent predictions across all time points. This is evident in Fig 2A with the number of PL participants correctly classified being less variable over time and the flatter gradients in the consistency of predictions in Fig 2B.

In Table 3, we show there is a much stronger signal discriminating PD from PL participants later in the disease course. This finding is expected as the PD participants, on average, will have a more severe disease at year 3 than they will at year 0. What these results therefore show is that by year 3 we have found a very accurate threshold for differentiating PD participants from PL. When we then back-propagate this threshold by testing the model over time, we find that the PL participants maintain a high predictive accuracy, but some PD participants cross this threshold and are misclassified as PL. As stated, differences between these groups can be largely explained by differences in their DNAm. Thus, we can attribute these findings to epigenetic modifications occurring in participants with PD as their disease progresses.

## 3 Discussion

In this paper, we applied an integrative network framework and artificial intelligence to the PPMI dataset. The PPMI dataset is an observational, international study, consisting of multiple data modalities, with the goal of identifying markers of PD to accelerate disease modifying clinical trials [17]. We used clinical, genomic, and proteomic data to include numerous patient samples and conducted cross-sectional and longitudinal stratification of participants who have PD, have an early indication of developing PD (PL), or were a HC.

We found that an integrative approach is optimal when performing disease stratifications for PD. Our models show a strong preference for including multiple modalities. It is clear that there is not adequate information in any one modality to accurately capture suﬃcient variability in PD at all time points. This highlights the importance of integrating multiple sources of information to capture different components of the heterogeneity in PD. Flexibility is also a key characteristic of this framework. Our approach allows us to test all modalities individually and all combinations of modalities at each time point. This allows us to perform ablation experiments to identify the most informative modalities at each time point.

The idiopathic subgroup contains individuals with no known cause or genetic association with PD. This makes them a very heterogeneous group as there could be a vast number of different disease mechanisms at play, which may not be captured by the clinical, genomic or proteomic data. It is therefore unsurprising that the accuracies achieved when integrating genomic data is lower, however our results show a disease signature is learnt when stratifying this subgroup. For this subgroup, our model identified CSF as very informative. Unlike whole-blood samples, that can only contain biomolecules that pass through the blood brain barrier, data derived from CSF likely contains a richer biomolecular complement that more closely mimics signatures in the brains of idiopathic PD participants. CSF was included in three experiments in the idiopathic subgroup, despite it only being available in a relatively low number of participants. Unfortunately, only one participant in the genetic subgroup had a CSF sample available. Thus, it is unknown if CSF is informative in the genetic subgroup of the PPMI dataset. As a result, it was not included in any genetic subgroup analyses and its effect was likely obscured in the joint genetic and idiopathic analysis. It is known that CSF is a good marker for PD as multiple CSF measures, in particular CSF *α*-synuclein, are known to be good prognostic measures of PD [26]. The build up of *α*-synuclein is well established in the pathology of PD, particularly later in the disease course, which mirrors our findings of CSF being more predictive later in the analysis [3]. It is still possible that there is a genetic cause of PD in this cohort and we also show that different genomics are informative at different stages of PD in the idiopathic subgroup. This supports the theory, by Wüllner et al. (2023), that the pathology or mechanisms of PD may change over time in this group and highlights the importance of flexibility when integrating different modalities [1].

There is a clear genetic driver in participants who have a mutation in a causal gene of PD. The genes of interest were *LRRK2*, *GBA* and *SNCA*, and are known to be associative with PD [3, 27]. The genetic influence on this group is far more prominent, and we show it can be distinguished with high classification accuracy using genomic data. A combination of DNAm and SNPs was identified as the most informative at all time points, highlighting a robust signal contained in the integration of these modalities. These results reflect the homogeneity between participants in this subgroup and the power of using a patient similarity approach.

There was a strong preference in all models for including DNAm irrespective of disease subgroup. DNAm was included in most optimal models in the cross-sectional experiments. This prominence indicates that DNAm is predictive of PD at all time points. Considering the importance of DNAm in both genetic and idiopathic groups separately and combined suggests that there could be an overlapping signal contained in this modality. As DNAm is a measure of epigenetics, it suggests that there is common environmental or behavioural factors in both genetic and idiopathic groups which explains some aspect of their PD. Further research to identify the main drivers of variability in DNAm in the two subgroups separately and combined should be conducted to identify these factors.

We show that training a model which integrates SNPs and DNAm later in the disease course could form a viable early diagnostic tool for individuals with a genetic association for PD. Genetic variance will be captured in the SNPs modality, thus making the HC participants easy to stratify. The variance which discriminates PD from PL is largely contained in the DNAm modality. DNAm is the process of binding methyl groups to sites in an individual’s DNA, resulting in alteration of expression [28]. It provides an epigenetic signature which can be inherited, associated with a disease and, depending on the site, reversed. Conditional to the DNA site affected, epigenetic modifications can occur slowly, meaning it can take a number of years for the effect of PD to be seen in a participant. The PPMI dataset does not take into account individual participant trajectories. For example, two participants with PD may be recruited and diagnosed at the same time but can have different disease courses. These facts mirror our findings that a better threshold for diagnosing PD is obtained later in the disease course. This is evident from our model, as we found the accuracy achieved when predicting the PL class is robust when testing the model at earlier time points. Conversely, there is a deterioration in classification accuracy of the PD group when testing the same model at earlier time points due to PD participants being misclassified as PL. Thus, by year 3, it appears the average disease state of a participant with PD in the PPMI dataset has progressed.

Diagnosing PD is a still an ongoing challenge of the disease, and being able to perform accurate early diagnosis would be a major step forward in its management. Diagnosis of PD in a clinical setting still involves the development of motor symptoms, by which time over 60% of dopamine neurons within specific regions of the basal ganglia may have been lost [29]. Pagan (2012) motivates that early detection can improve outcomes for PD patients by slowing disease progression and limiting its effect on their quality of life [29]. In the PPMI study, genomic data was generated using whole-blood samples from participants. The advantage of using whole-blood samples is that they are minimally invasive and cost-effective. The disadvantage is that the biological signal may be quite weak for a neurological disorder in the blood due to the blood-brain barrier. Despite these limitations, we have shown excellent accuracy at all time points, making this a promising and viable approach to develop an early diagnostic tool for PD.

There are limitations to the model presented in this analysis. It is preferable that the sensitivity of the PD class rather than the specificity be accurate, as is the case here. If the sensitivity is high it means that the model is more likely to misdiagnose a PL participant as a PD which is preferable to misdiagnosing many PD participants. It cannot be determined how accurate this model is prior to a clinical PD diagnosis. This analysis is limited by the longitudinal time points of the PPMI dataset. Tracking the accuracy of this model for PL participants who go on to develop PD is a promising avenue of future research to further develop an early diagnostic tool. Further research also needs to be conducted in a dataset other than the PPMI dataset to measure the robustness of these findings. There is potential for survivor bias in the participants included in the longitudinal analysis due to the use of a GCN model. A GCN is a transductive graph neural network algorithm, meaning all nodes have to be present during training and testing [30]. As a result, all participants are required to be present at each time point in order to be included in this longitudinal analysis. This leads to potential survivor bias, as participants will have survived the disease until at least year 3 of this analysis. Future implementations should look towards inductive graph neural network algorithms which do not require all nodes to be present during training. This will allow us to train models on unseen datasets and include all participants at each time point, thus eliminating survivor bias.

## 4 Conclusion

This study highlights the importance of flexible integrative approaches to the analysis of PD. We have shown that there is a signal for PD present in genomic and proteomic data obtained from whole-blood samples. We have shown this both in a homogeneous group with a clear genetic driver for the disease and also in a more heterogeneous idiopathic group. We have achieved non-zero improvements in accuracy which are comparable to the MDS-UPDRS assessment baseline in the idiopathic group and significantly improved on this baseline in the genetic group. We have done so with models that do not account for the effects of medication or individual PD participant trajectory. We have identified DNAm as an informative omic measure in all individuals with PD and have proposed a model which could be used as an early diagnostic tool for individuals with a genetic predisposition for the disease. In summary, our research shows that an integrative network framework can be used to perform longitudinal stratification in PD.

## Supporting information

S1 Fig Graph convolutional network - multi-modal encoder architecture (GCN-MME). The GCN-MME takes as input a fixed network and any number of modalities. The nodes in the network correspond to patients, and each patient is present in at least one modality. The modalities are encoded for dimensionality reduction using a two layer encoder. After the second layer, median imputation is performed to include patients missing from that modality but included in the network and at least one other modality. There is a shared latent embedding between the encoders, and the imputed second layers of each encoder are joined using mean pooling. This shared latent embedding forms the node features for the GCN. Patient classification is performed using the GCN with the loss back propagated through the entire GCN, thus, training each encoder in series with the GCN. (TIF)

S2 Fig Breakdown of clinical diagnosis criteria and disease subtypes. (TIF)

S3 Fig Confusion matrix for best MOGDx models from idiopathic and genetic combined subgroups at each time point. (TIF)

S4 Fig Confusion matrix for best MOGDx models from genetic subgroup at each time point. (TIF)

S5 Fig Confusion matrix for best MOGDx models from Idiopathic Subgroup at each time point. (TIF)

S6 Fig Confusion Matrix for MOGDx model trained at Year 0 and tested at all time points. (TIF)

S7 Fig Confusion Matrix for MOGDx model trained at Year 1 and tested at all time points. (TIF)

S8 Fig Confusion Matrix for MOGDx model trained at Year 2 and tested at all time points. (TIF)

S9 FigConfusion Matrix for MOGDx model trained at Year 3 and tested at all time points.(TIF)S1 Table. Optimal latent dimension embeddings per modality.

**Table 4:** 

MME Modality Reduced Dimension						
mRNA	miRNA	DNAm	CSF	Clinical	SNP	MDS-UPDRS
32	16	32	16	4	8	8

S1 File Supplementary file of all model experiment results. (CSV)

S2 File Supplementary description of MOGDx model. (PDF)
