# Supplementary figures and images for: An integrative network approach for longitudinal stratification in Parkinson’s disease
Source: PLoS Comput Biol. 2025 Mar 28;21(3):e1012857. doi: 10.1371/journal.pcbi.1012857 (PMC11957384; doi:10.1371/journal.pcbi.1012857)

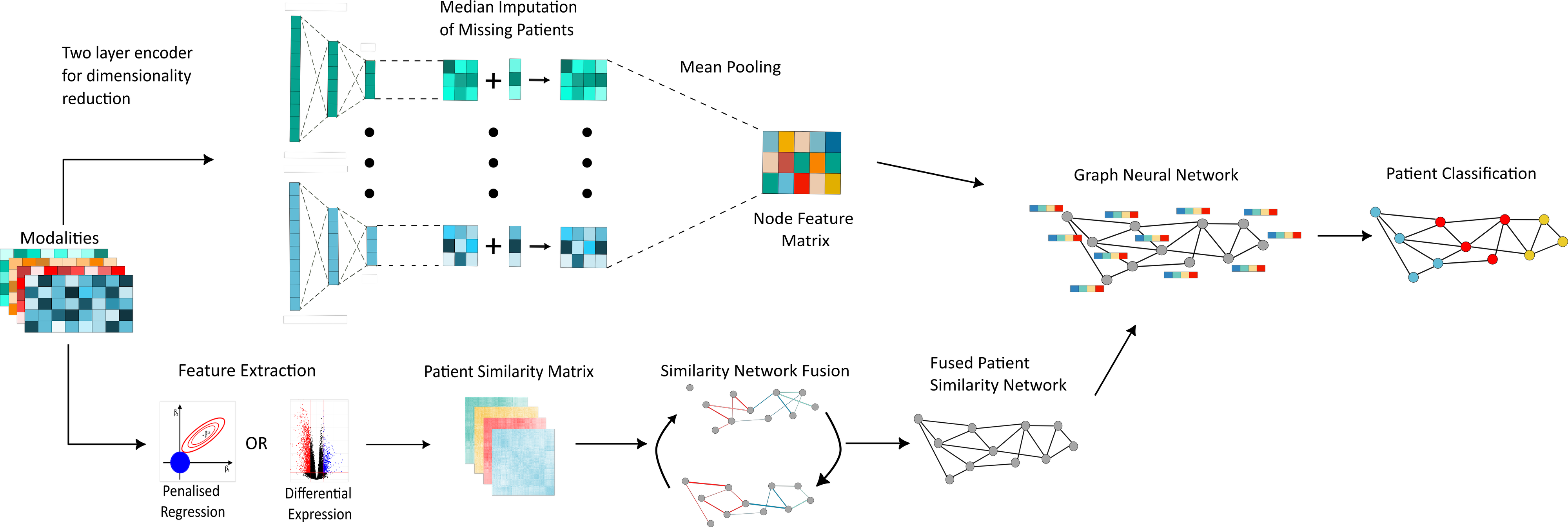

Supplement: S1 Fig — (TIF) [file pcbi.1012857.s001.tif]

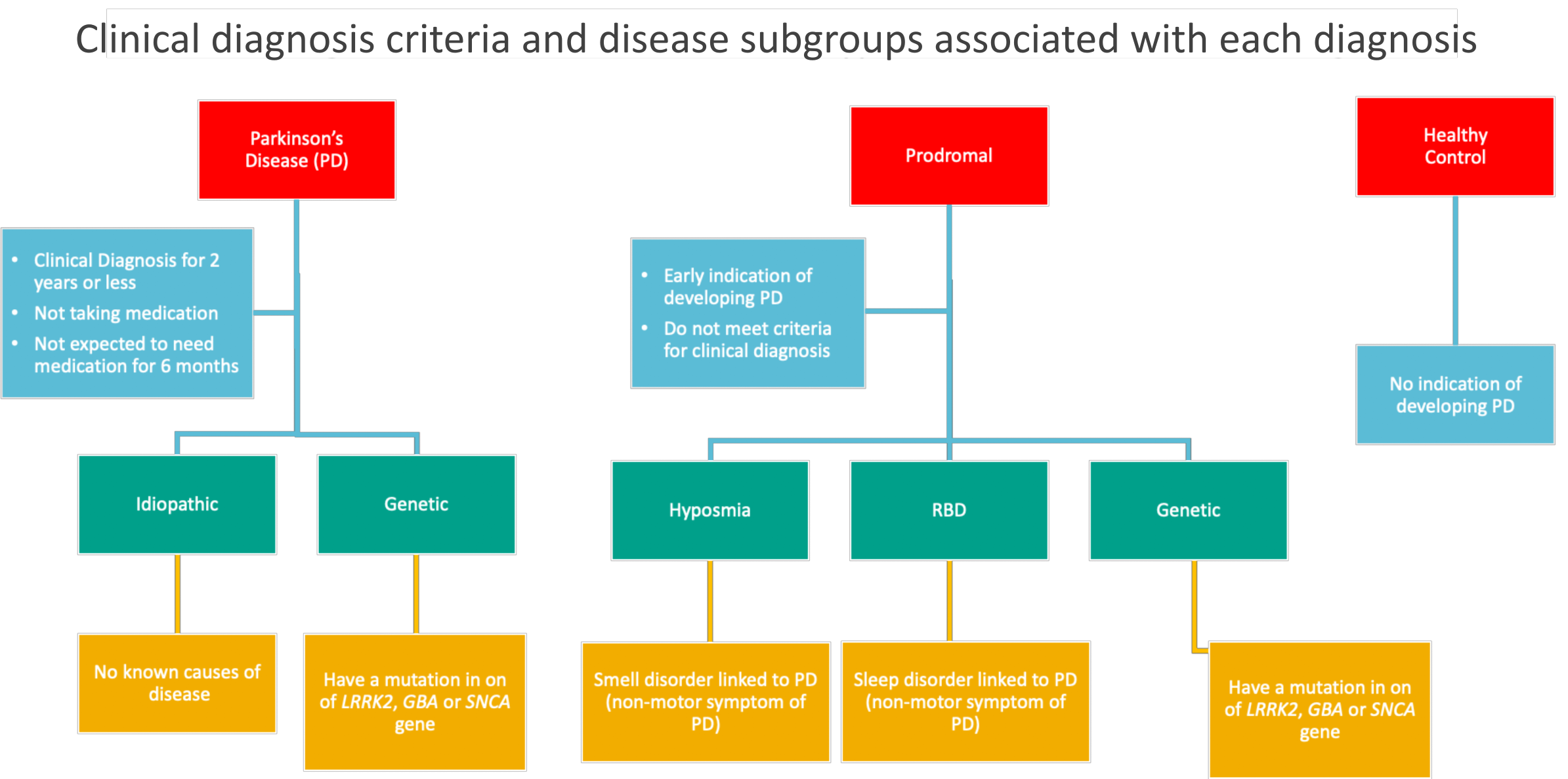

Supplement: S2 Fig — (TIF) [file pcbi.1012857.s002.tif]

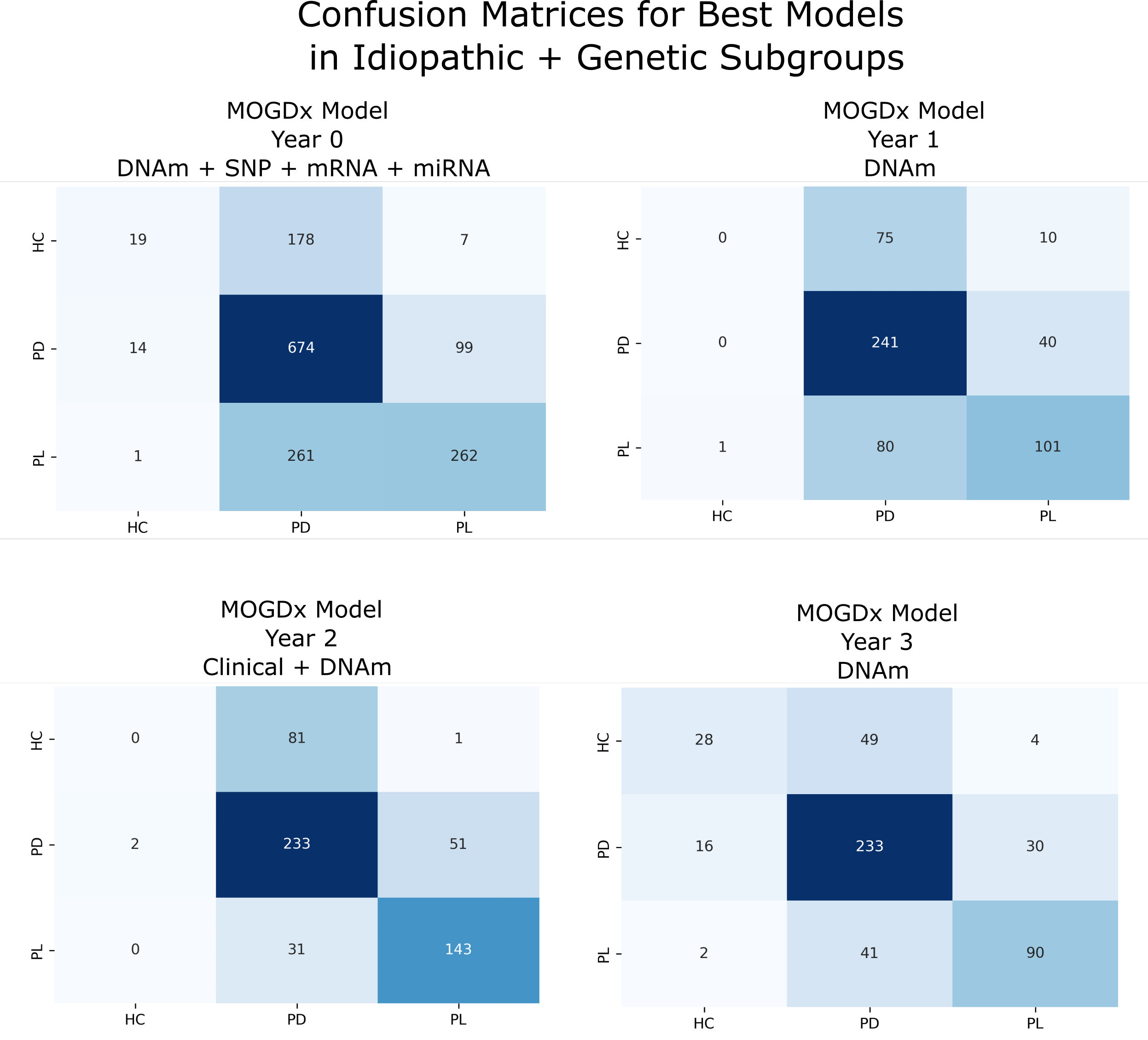

Supplement: S3 Fig — (TIF) [file pcbi.1012857.s003.tif]

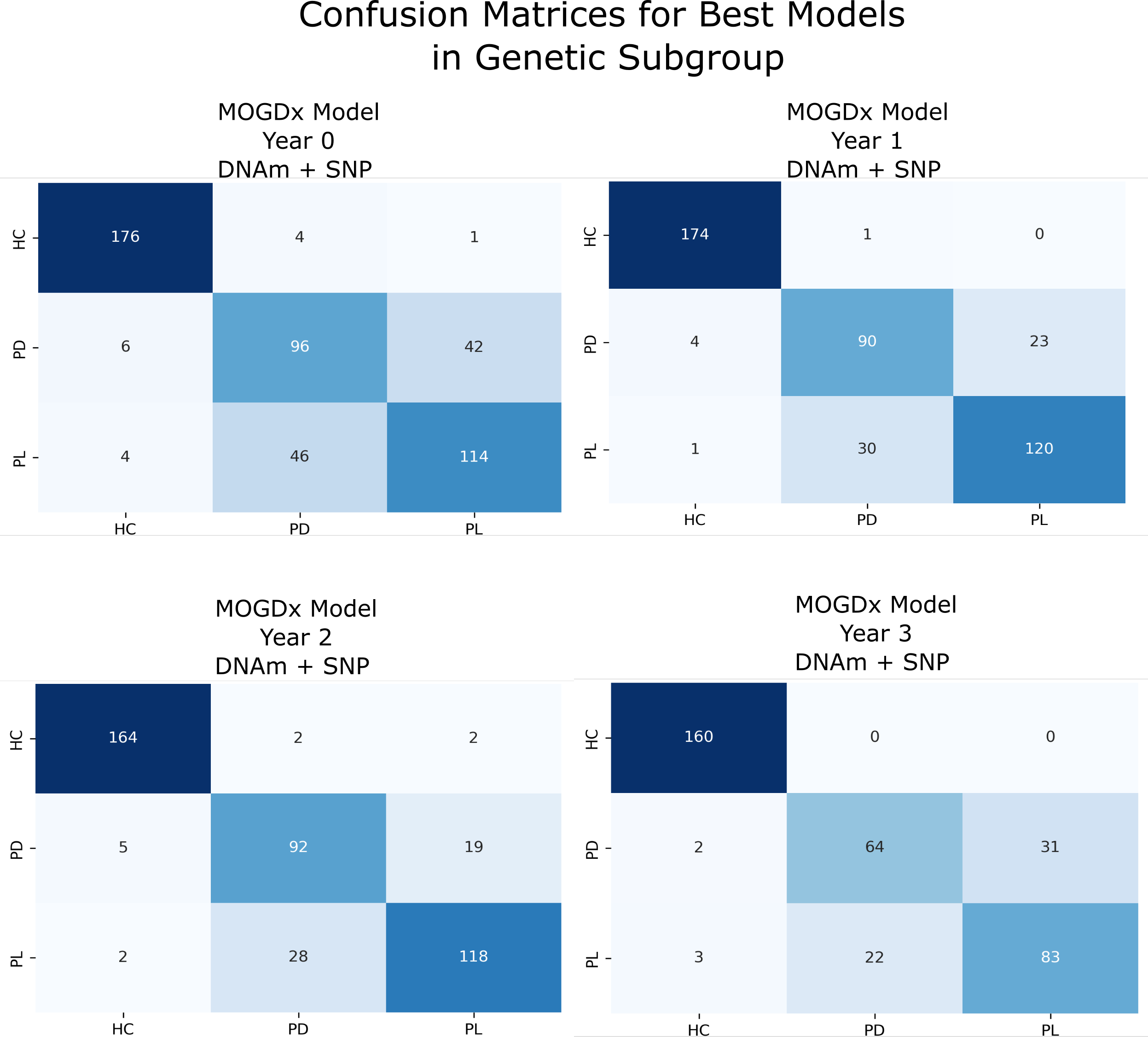

Supplement: S4 Fig — (TIF) [file pcbi.1012857.s004.tif]

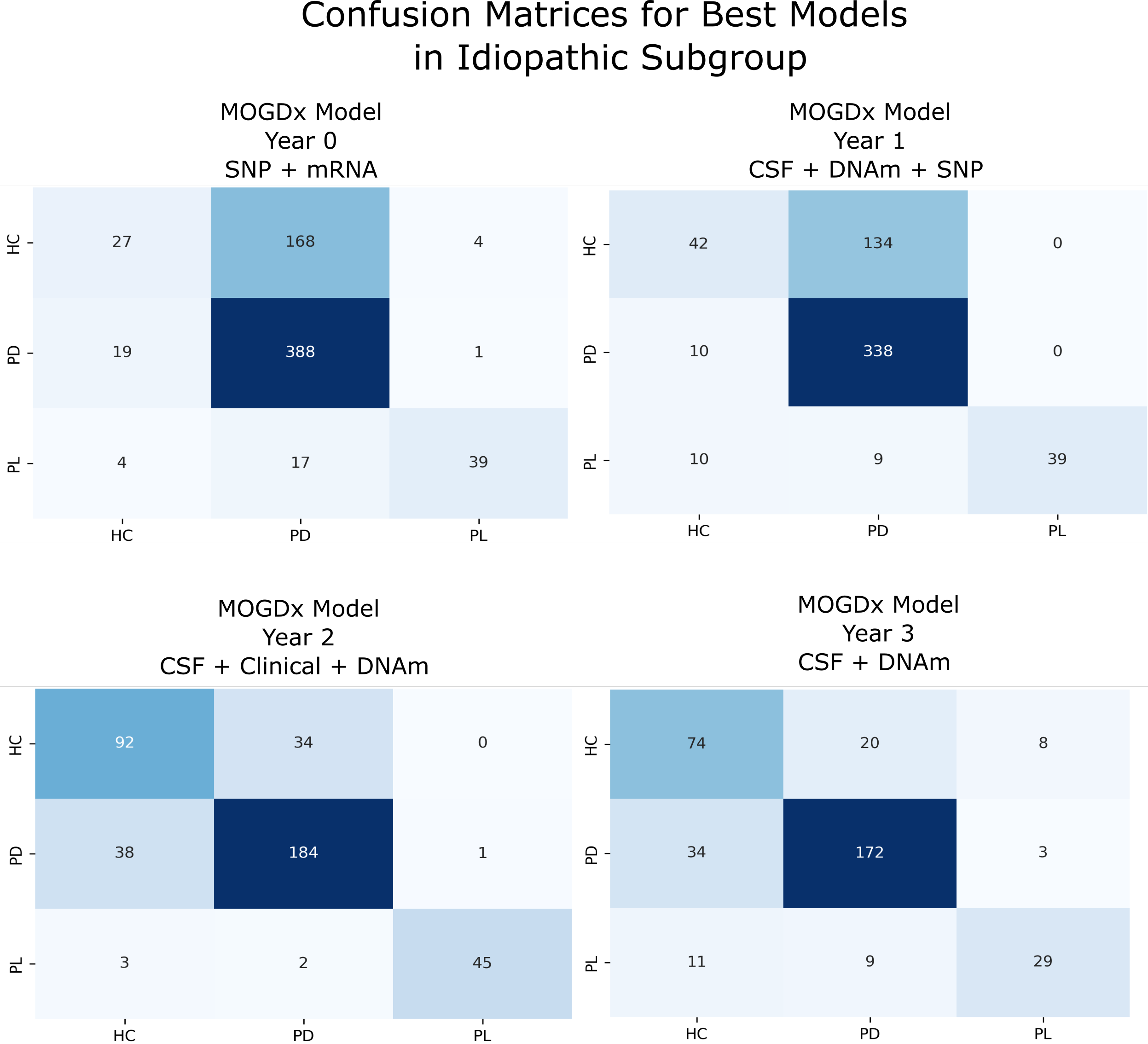

Supplement: S5 Fig — (TIF) [file pcbi.1012857.s005.tif]

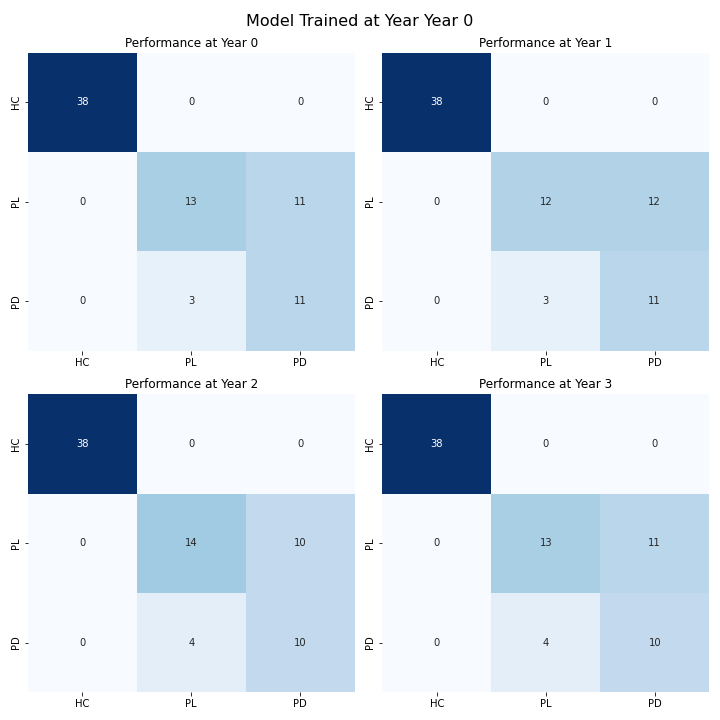

Supplement: S6 Fig — (TIF) [file pcbi.1012857.s006.tif]

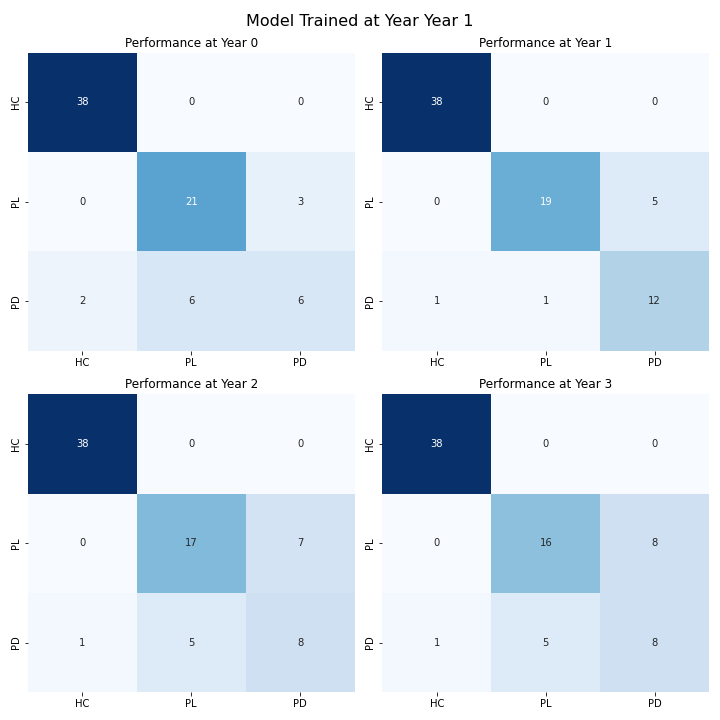

Supplement: S7 Fig — (TIF) [file pcbi.1012857.s007.tif]

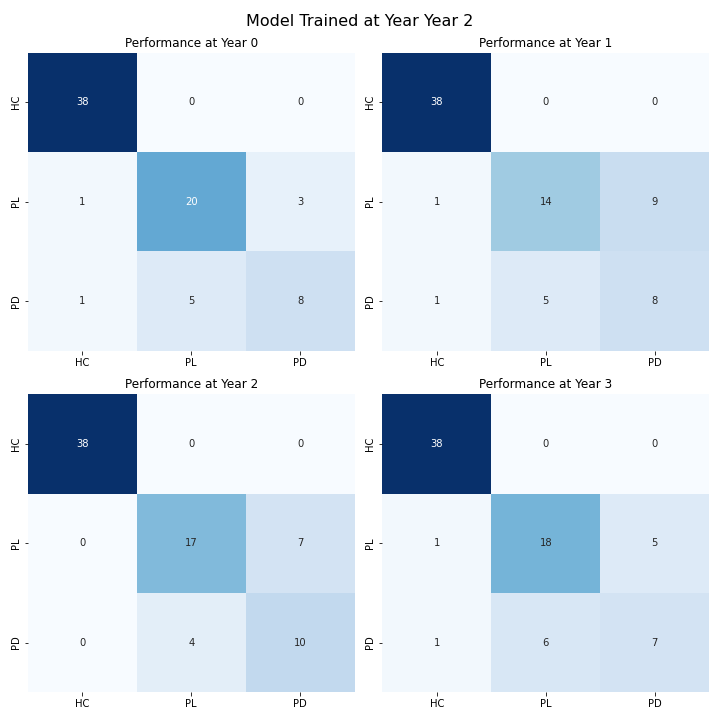

Supplement: S8 Fig — (TIF) [file pcbi.1012857.s008.tif]

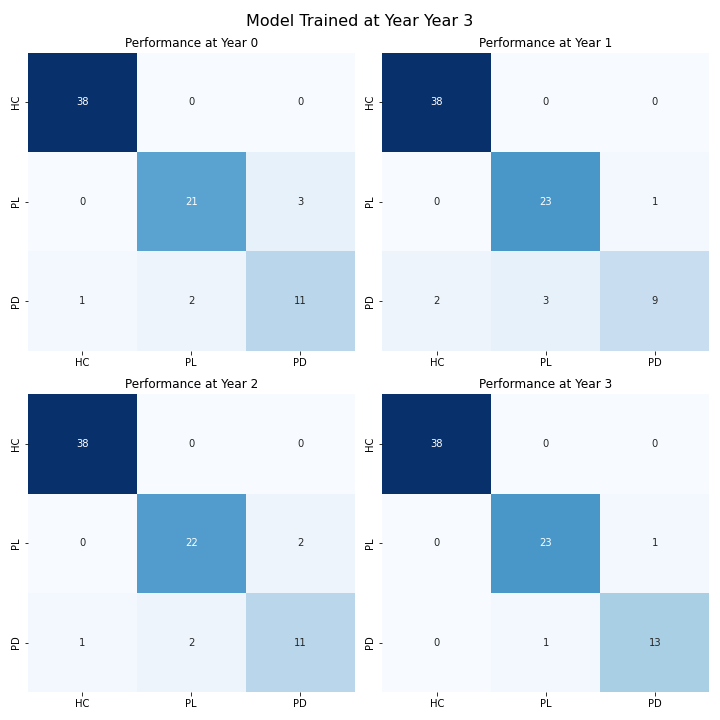

Supplement: S9 Fig — (TIF) S1 Table. Optimal latent dimension embeddings per modality. [file pcbi.1012857.s009.tif]
