# Supplementary material for: An integrative network approach for longitudinal stratification in Parkinson’s disease
Source: PLoS Comput Biol. 2025 Mar 28;21(3):e1012857. doi: 10.1371/journal.pcbi.1012857 (PMC11957384; doi:10.1371/journal.pcbi.1012857)
Supplement: S2 File — (PDF) [file pcbi.1012857.s011.pdf]

Supplementary Description of Model

The model employed is Multi-Omic Graph Diagnosis (MOGDx) or MOGDx. Its architecture consists of three main parts; a Multi Modal Encoder (MME), a fused Patient Similarity Network (PSN) and a Graph Convolutional Network (GCN), with the architecture shown in S1 Fig. The MME consists of two linear layers. First, each modality is encoded to a reduced linear layer of dimension 500. Batch normalisation is performed and the output of this linear layer is encoded further to a second linear layer of arbitrary dimension. The output dimension of the encoder is specific to each modality, was found through hyperparameter search, and is shown in Table 4. Further batch normalisation and median imputation of missing patient samples on this encoded layer are performed. The encoded output of each modality is decomposed into the shared embedding space through mean pooling.

A PSN is generated per modality and fused using similarity network fusion leaving a single network representing similarity between patients across multiple modalities. Each node in the fused PSN corresponds to a single patient who has a corresponding vector from the MME shared embedding space.

We provide the GCN with two inputs; our fused PSN and the MME embedding space. The GCN was trained on 5 random cross validated splits of the data, with both the GCN and MME trained on the same data splits. Each model is trained for 2000 epochs, with early stopping implemented if the model performance has not improved in the previous 250 epochs. Model parameters such as learning rate were previously validated through hyperparameter searches and fixed for all models trained in this analysis. See Ryan et al. for more detail[1].The weights of the MME are learnt jointly with the GCN by evaluating the loss on the GCN making it a fully supervised pipeline. The MME and GCN embedding space dimensions are found through hyperparameter searches and provided in Table S1. When training the network, within each data split, nodes are divided into training nodes and transductive test nodes. These transductive test nodes are not included in the loss computation, but they are still involved in the message passing algorithm[2]. This methodology means that although the labels are hidden, the graph does use test data to update its embedding representations. This methodology differs to classical supervised learning setting, in which the test data is removed from the model and is referred to as the semi-supervised training of GCN[2,3].

Bibliography

1. Ryan, B., Marioni, R. E. & Simpson, T. I. Multi-Omic Graph Diagnosis (MOGDx): a data integration tool to perform classification tasks for heterogeneous diseases. *Bioinformatics* **40**, btae523 (2024).
2. Hamilton, W. L. *Graph Representation Learning*. (Springer International Publishing, Cham, 2020). doi:10.1007/978-3-031-01588-5.
3. Kipf, T. N. & Welling, M. Semi-Supervised Classification with Graph Convolutional Networks. Preprint at http://arxiv.org/abs/1609.02907 (2017).
